# Design of the Automated Calibration Process for an Experimental Laser Inspection Stand

**DOI:** 10.3390/s22145306

**Published:** 2022-07-15

**Authors:** Jaromír Klarák, Robert Andok, Jaroslav Hricko, Ivana Klačková, Hung-Yin Tsai

**Affiliations:** 1 Institute of Informatics, Slovak Academy of Sciences, 845 07 Bratislava, Slovakia; robert.andok@savba.sk (R.A.); hricko@savbb.sk (J.H.); 2 Department of Automation and Production Systems, Faculty of Mechanical Engineering, University of Zilina, 010 26 Zilina, Slovakia; ivana.klackova@fstroj.uniza.sk; 3 Department of Power Mechanical Engineering, National Tsing Hua University, Hsinchu 30013, Taiwan; hytsai@pme.nthu.edu.tw

**Keywords:** automation, Fourier transform, laser sensor, calibration, scanning, Industry 4.0

## Abstract

This paper deals with the concept of the automated calibration design for inspection systems using laser sensors. The conceptual solution is based on using a laser sensor and its ability to scan 3D surfaces of inspected objects in order to create a representative point cloud. Problems of scanning are briefly discussed. The automated calibration procedure for solving problems of errors due to non-precise adjustment of the mechanical arrangement, possible tolerances in assembly, and their following elimination is proposed. The main goal is to develop a system able to measure and quantify the quality of produced objects in the environment of Industry 4.0. Laboratory measurements on the experimental stand, including the principal software solution for automated calibration of laser sensors suitable for gear wheel inspection systems are presented. There is described design of compensation eccentricity by Fourier transform and sinusoidal fitting to identify and suppress the first harmonic component in the data with high precision measuring.

## 1. Introduction

Gear wheels are the principal components of many mechanical devices for transmission and reduction of mechanical energy. Considering the implementation of Industry 4.0, the mass production of gear wheels poses high demands for inspection and quality evaluation of products. The specific form of wheels containing the involute geometry of teeth imposes serious problems for inspection tasks of these form-complex objects. Several inspection methods have been developed recently. The optical inspection with camera vision [1] detects the internal hole and number of teeth. The vision-based inspection [2] with deep learning image processing enables the detection of surface defects. The survey of approaches based on the camera sensing and processing data by Deep learning methods is discussed in [3]. The measuring system for sensing flexural motions of compliant mechanism based on the distance laser sensor and the camera vision is presented in [4]. Basic calibration processes use camera vision for object recognition implementing an IRVision (FANUC company) system based on a two-dimensional dot grid with the definition of the calibration points described in [5]. This method is a commercial system dedicated to camera vision in industrial robotic applications. In [6], a calibration method of triangulation scanner is described, which is used to map the 3D surfaces of blades. These are very specific according to their shape. In distances of 250 to 650 mm, an accuracy of 1 mm is declared. Specific measuring systems and calibration methods are used in the aeronautics and astronautics industry. This work describes using a laser distance sensor mounted on a CMM measurement machine to scan the metallic sphere mounted on the rotary platform with the measuring error declared to be 0.05 mm [7]. The calibration process for the robotic arm used with the laser sensor is described in [8]. The main goal of this work was to demonstrate the accurate positioning of the robotic arm according to simulations. The system calibration is based on iterative and optimization solvers. The work compares values from simulation and calibrated and non-calibrated measurements, respectively. The measuring system with included calibration is the principal requirement for the good functioning of measuring machines [9].

The design of any mechanical and other hardware can lead to serious errors when precise measurements are required. For this reason, further works focused on developing calibration methods using camera vision or vision combined with the dot and line lasers are needed [10,11]. In the case of the calibration process, many papers already published focus on robot calibration, as discussed for instance in [12], where a method using sensor Microepsilon scanControl 2600–100 for the entire system calibration and reducing mean planar errors from 530 μm to around 230 μm is proposed. Similar work with laser sensor by Micro-Epsilon used for robot calibration is described in [13]. Calibration of laser sensors in an automated production line is proposed, e.g., in [14], where a solution with absolute accuracy of ±500 μm for positioning of large antennas of cca. 12 m × 2.5 m dimensions is described. Another work describes an application of the distance laser sensors in measuring the pieces inside the workplace of a CNC machine named “laser on-machine measurement” (LOMM). There the calibration process is described using rotation matrices. The measurements by the displacement sensor are compared to a touch probe. A difference of 4.7μm of the calibrated system to an actual sphere center after five iterations was reached [15]. The work using the laser sensor from KEYENCE company (type J-V7060) describes the device for reverse engineering. There are also calibration processes mentioned based on rotation and translation matrix. For the calibration procedure, a sphere was used [16].

In the practical application of measurements in industry, mainly in factory environments, are used devices, for example by Renishaw company, where these devices serve for calibration mainly of CNC machines (for instance device Ballbar or Laser interferometry). The main advantages of these devices are remarkable precision and measurement resolution. From a wide range of applications, only specific applications are for one-axis measurement (Laser interferometry) or measurement in 2 axes (Ballbar), which is not suitable for more general measurement purposes such as area scanning, 3D scanning, etc. [17,18,19]. In the market and industry, is it possible to meet more commercial devices which are, in principle, very similar to the products from the Renishaw company.

In the case of a calibration process with the removal of the first harmonic component as a sine wave, it is necessary to correctly identify the basic parameters, namely the frequency of the harmonic component, the amplitude corresponding to the eccentricity of the shaft fixation, and the phase shift. These parameters can be identified by fitting the sine wave to the data. There are several methods proposed in professional publications depending on the nature of the data, computational power, and their purpose of use. The most common application is using the least mean square error method, for instance, sine fitting with the 4-parameter method [20] or sine fitting method and its comparison with conventional methods [21].

This article describes a solution based on several methods, where the Four-Parameter Case part is solved by an iterative counting method. Other methods of sinusoidal wave fitting are given in [22], where the authors calculated the error through iterations, or in [23], where a sine fitting solution with discrete values is described. In general, these methods for fitting sine waves are proposed. Still, their generality is based on counting errors in iteration cycles, or their accuracy is estimated in percent of the range of the dataset used.

For the measuring and inspection applications in modern production environments, calibration processes are crucial and necessary to reach acceptable measuring accuracy and high-quality inspection. The calibration is performed before every new setting of the system. Therefore, automation of highly complex processes such as calibration would remarkably reduce pre-manufacture times for more intelligent systems that would continue being developed in the new areas of Industry 4.0.

## 2. Inspection System Proposal

### 2.1. Problem Analysis and Concept

**Figure 1:**
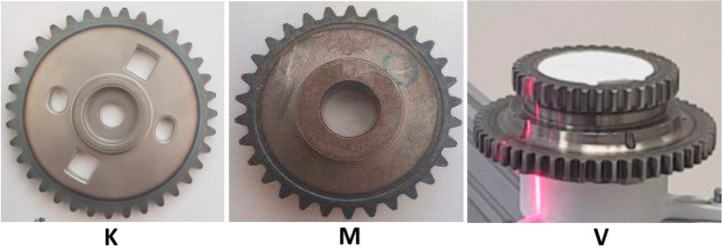
Illustration of various gear wheels (types “K”, “M”, and “V”) as objects of inspection.

As an object of inspection, a gear wheel is used (Figure 1) that represents the number of mechanical elements of mass production. The aim of this paper is to collect and process data to generate the point cloud corresponding to the real geometry of the gear wheel. The final goal is preparing necessary data for evaluation of the production quality and further automation of the system towards to zero-defect production process [24]. As illustrated in Figure 1, there are many different shapes of gear wheels in industry. For every type of gear wheel, a unique setting of the inspection stand is necessary for obtaining appropriate data.

**Figure 2:**
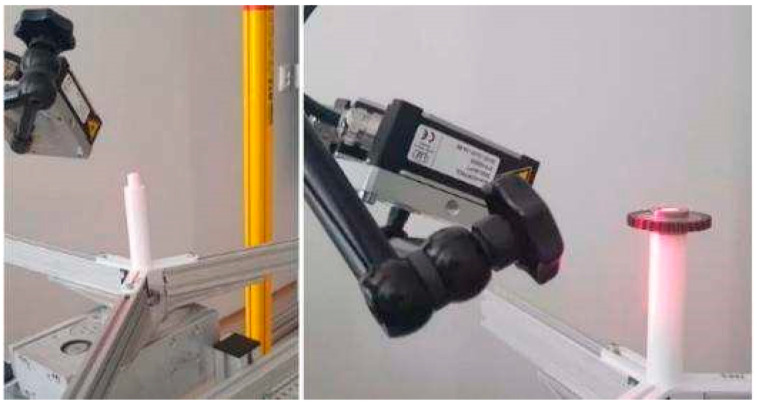
The experimental inspection laboratory stand; first step (**left**): scanning the shaft as a calibration process; second step (**right**): scanning the gear wheel.

Following previous studies and work [10], laser sensing was chosen as the most suitable measuring principle for the inspection. For basic laboratory experiments, an experimental inspection stand was set up, illustrated in Figure 2, which was built upon a universal rotation table with angle position control. In this stand, a shaft and laser sensor were used. Parameters of the mentioned sensor are in Table 1 [19].

Numerous experimental measurements on several conventional objects have been performed. Obviously, measurement accuracy strongly depends on the precise positional adjustment of sensors concerning the object. To achieve precise measurement, a calibration procedure is required to eliminate errors in the fixation of sensors, tolerances of parts dimensions, backlashes, or possible misalignments. For this purpose, the software calibration procedure is proposed and described in Section 2.2. (Methodology of calibration procedures) and in Section 2.3. (Data preparation). Thus, the measuring stand can easily adapt to real objects or situations.

Due to the number of different types of gears produced, it is necessary for each type to define the specific position and orientation of the laser sensor in relation to a particular scanned gear. Based on the initial analysis and experiments with a possible inspection system, the main areas requiring to be addressed have been identified:

1. Adapting the position of the laser sensor according to the gear wheel regarding the specifics of the scanned object and its functional surfaces to be inspected.

- The main fundamental is to design a cognitive solution for transforming data from the coordinate system of the laser sensor, specifically located in space, to the coordinate system of the shaft as the first step of calibration.

2. The second calibration step is to solve the accuracy of the measurements by suppressing the eccentricity of the shaft position.

- The method used is based on applying the Fourier transform in the first step, followed by developing a precise fitting of the harmonic component.

### 2.2. Methodology of Calibration Procedures

**Figure 3:**
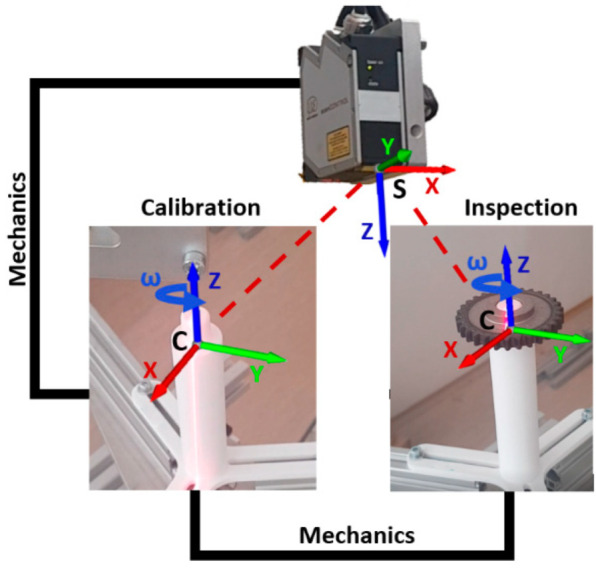
The reference systems for calibration: S—Sensor (unprocessed point cloud), C—fixation shaft.

The goal of the calibration procedure is to find the exact positions of the rotary shaft with respect to a particular sensor and the actual configuration of the measuring system. The positional errors due to non-precise fixation of sensors, inaccuracies of manufacturing of pieces, assembly errors of the stand, or eccentricity in a rotation table with a fixation shaft, result in measurement errors and deteriorate the accuracy of inspection apparatus. For calibration, we state two rectangular reference coordinate systems, as shown in Figure 3:

- S(x,y,z) the sensor reference system in which measured values/data are given;
- C(x,y,z) is the system related to the fixation shaft as the reference for the position of an object (gear wheel).

The S(x,y,z) reference rectangular system is a basic sensor coordinate system, where all scanned points (and their coordinates in X, Y, and Z) are defined in this reference system. Due to unifying data modified point cloud is necessary from the laser sensor and unifying scans to C(x,y,z) rectangular coordinate system, which is concatenated to the shaft. Let’s state that point $\vec{P}$ on the object surface captured by a sensor in the S reference system is denoted by the displacement vector $\vec{D}$ and transform matrix **O** to C reference system. The position of this point P expressed as the vector $\vec{P_{c}}$ in C-reference system is then using transformation (1):

(1) $$\vec{P_{c}}=\vec{D}.O.\;\vec{P_{s}}$$

where **O** is (3 × 3) transformation matrix between particular reference systems. Once the configuration of an inspection stand, i.e., mechanics, with a dedicated object and sensory system arranged, the sources of errors emerge mainly in mechanics. To guarantee maximal sensing accuracy, it is necessary to find the exact transformation between the S and C reference systems. Thus, the principal problem of calibration is to reveal and identify errors that arise between references S and C and the transformation change between both systems. This naturally should be done for each sensing position of the table/wheel, concerning scans in rotation, as well. To eliminate these estimated errors, one can include error corrections in the form of a corrective matrix **E**. Then, any measurements of position revised $\vec{P_{ce}}$ in the C reference system can be calculated according to relation in (2):

(2) $$\vec{P_{ce}}=E.\;\vec{P_{c}}$$

### 2.3. Data Preparation

**Figure 4:**
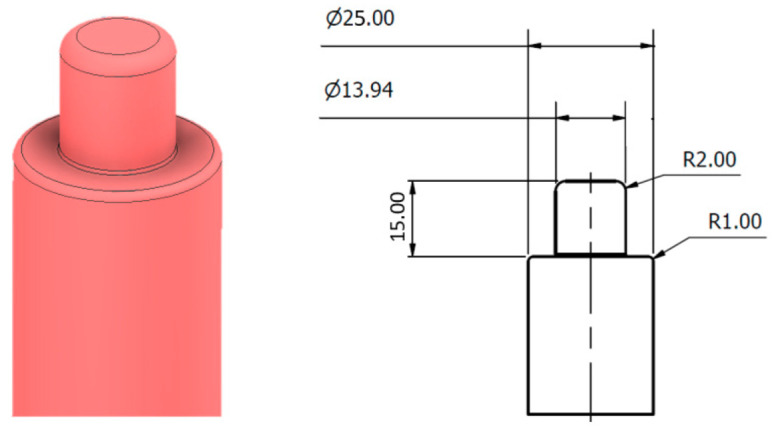
Shaft for gear wheel type: M.

The calibration basis is the scanning of the rotating shaft around the axis and the expression of these points in the S-reference coordinate system. The dimensions of the sensing shaft are shown in Figure 4. This shaft consists of two basic parts. A basic cylindrical surface with a diameter of Ø25 mm and a bearing cylindrical surface with a nominal size of Ø13.94 mm, which are scanned by a laser sensor.

**Figure 5:**
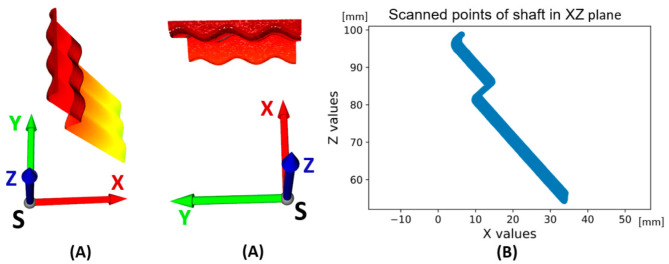
Data visualization of scanned shaft surfaces, measuring 1, file “k1.csv” (**A**)—3D, (**B**) scanned surfaces projected to 2D—plane XZ.

Based on the system shown in Figure 2 and Figure 4, the points captured by the laser sensor and representing the surface of the sensed shaft in the coordinate system S are generated. The result is the unfolded area of the sensed shaft shown in Figure 5A. The whole image consists of a sequence of scanned profiles in the X and Z axes (XZ plane) in time, which is expressed by the Y axis. All scanned profiles captured by the sensor in the XZ scanning plane are shown in Figure 5B. This makes it possible to express each scanned point by the vector $\vec{P_{s}}$ as $\vec{P_{s}}=\left[X_{s},Y_{s},Z_{s}\right]$. The basic parameters of shaft sensing are given in Table 2. The scanned shaft data were stored in a .CSV file under a name consisting of k—calibration and scan number, i.e., “k1”.

Based on Equation (3), it is necessary to define parameters for performing the transformation of points from the coordinate system S to the coordinate system C and to correctly generate values for the vector $\vec{D}$ and for the matrix **O**:

(3) $$\;\vec{D}=\left[\begin{array}{ccc} f\left(x\right) & 1 & f\left(z\right) \end{array}\right]$$

(4) $$O=\left[\begin{array}{ccc} \cos\varphi & 0 & -\sin\varphi \\ 0 & 1 & 0\\ \sin\varphi & 0 & -\cos\varphi \end{array}\right]$$

The displacement vector can be expressed as a correction of the point values in the X and Z axes, being basic scanning axes of each profile. The goal is to get the scanned points to the starting position and to ensure the unification of the shaft axis with the Z axis in the C coordinate system. Three-dimensional matrix **K** consists of three two-dimensional matrices for each axis **X**, **Y,** and **Z**, as given in Equation (5). The two-dimensional matrix in size i, j for each axis is in the dimension 640 × 11,000, which corresponds to the sensing based on the possibility of the sensor used. So, 640 corresponds to the maximum number of scanned points in one profile (Table 1—Scanning points) and the number of scanned profiles in one scan, which in this case is 11,000 profiles, according to Table 2.

(5) $$K=\left[\begin{array}{ccc} X & Y & Z \end{array}\right]$$

**Figure 6:**
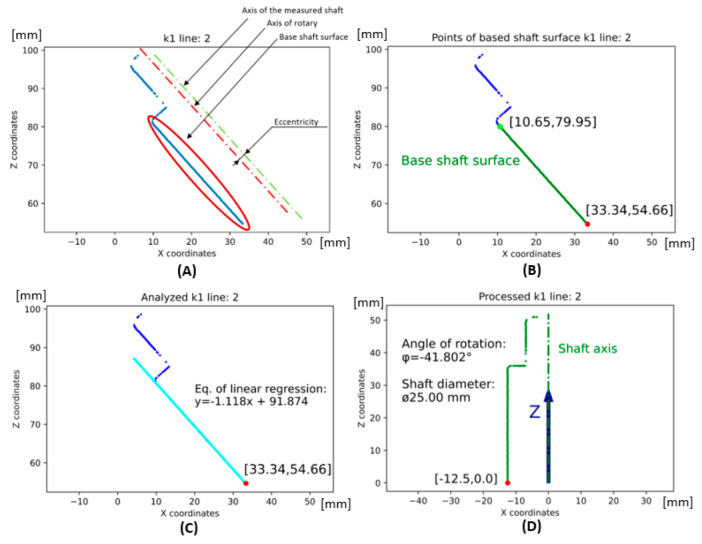
(**A**) Line scan XZ—axis in S-reference system, (**B**) definition base shaft surface, (**C**) linear regression according to base shaft surface, (**D**) transformation of point cloud into C-reference system.

The displacement vector is expressed as the displacement of the points in the X and Z axes, the displacement value for the Z axis corresponding to the lowest value in the **Z** matrix of the **K**-matrix (scan). The dot is shown as a red dot in Figure 6B–D.

(6) $$\vec{D}=\left[\begin{array}{ccc} X_{i,j} & 1 & \min\left(Z\right)) \end{array}\right]$$

**Figure 7:**
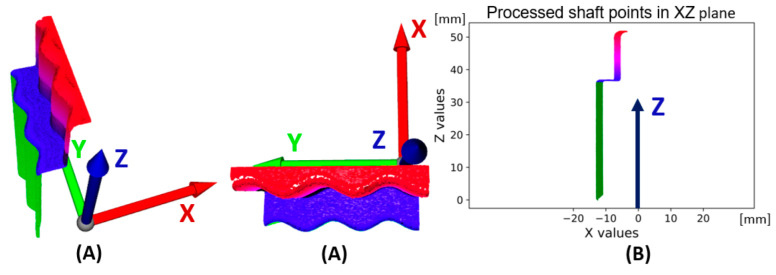
Data visualization of processed shaft surfaces scan, measuring 1, file “k1.csv” (**A**)—3D (green surface—Base shaft surface), (**B**) scanned surfaces projected to 2D—plane XZ (green line—Base shaft surface).

The second process is to perform a transformation of points around the zero point of the C coordinate system. The basis is to use the transformation matrix given in (4). The angle φ is expressed as the angle between the X axis of the coordinate system and the axis of the shaft, respectively, of a cylindrical surface with a parallel axis of symmetry. There are two cylindrical surfaces in the scanned shaft. The larger cylindrical surface is the Base shaft surface shown in green, where the coordinates of the endpoints of this surface are also shown in Figure 6B. The points from the Base shaft surface are used in the form of a one-dimensional matrix in the polynomial regression shown in Equation (7) and displayed in cyan color in Figure 6C. Subsequently, the angle is calculated according to Equation (8) as the arc tangent of the parameter k (gradient) from the directional expression of the line according to Equation (7) in two-dimensional space. The numerical expression of the angle between the line and the Z axis is expressed in Figure 6D as −1/2π − φ in the final value rounded to 3 decimal places: −41.802° (deg). A 3D representation of the point cloud ($\vec{P_{c}}$) processed according to Equation (1) is shown in Figure 7A. The projection of all points into the XZ plane is shown in Figure 7B. The sinusoidal character of the recorded points can be seen in these illustrations. This is due to the so-called measurement error, namely the eccentricity of the location of the rotating shaft that is sensed and the rotating table. Ideally, the axis of the shaft should be in line with the axis of the rotating table, which is not the case and manifests itself as the first harmonic component in the measurement:

(7) $$Y={\left(X_{g}^{T}X_{g}\right)}^{-1}X_{g}^{T}C_{x}\;$$

(8) φ=arctan(k)

**Figure 8:**
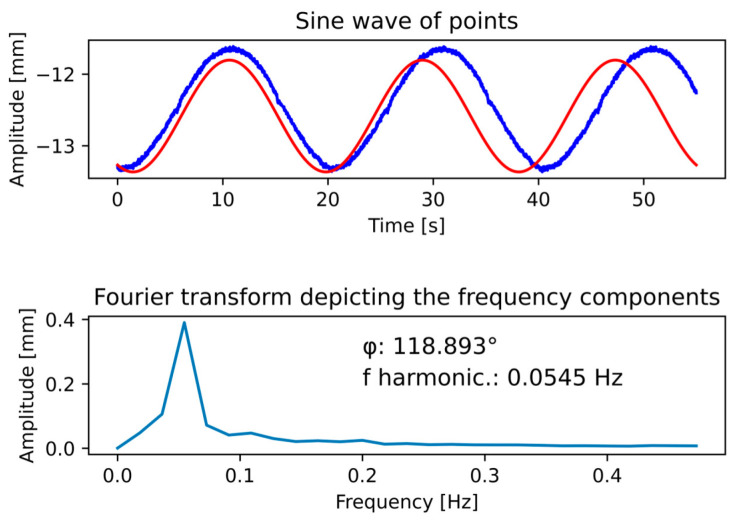
Fourier transform according to eccentricity sinusoidal wave; above: blue—*z* line in plane XY corresponding to amplitude and time, red—computed sinusoidal wave based on Fourier transform; below: plot of harmonic frequencies and their amplitudes.

The most common solution for removing the first harmonic component from the measurements can be performed using the general discrete Fourier transform suitable for the point cloud given in (9), where *N* declares the number of samples—in this case, 11,000 (according to Table 1), *n* means evaluation, *k*—frequency, and X(k) mean $k^{\mathrm{th}}$ frequency. The graphical output of the Fourier transform is shown in Figure 8. In the upper part of the figure, the course of the sinusoidal character relation between the points according to time (Y axis) and the amplitude of the points (X axis) is displayed in blue. The resulting harmonic frequencies with amplitudes are shown at the bottom of Figure 8, where the first harmonic component was expressed as the real part for a particular harmonic frequency. The subsequent phase shift φ is expressed as the value of the imaginary component from the Fourier transform at a particular harmonic component. A comparison of the real points and the calculated first harmonic component is shown in Figure 8 above (red sine wave).

(9) $$X\left(k\right)=\frac{1}{N}\sum_{n=0}^{N-1}\left(x_{\left(n\right)}e^{-j\frac{2\pi }{N}nk}\right)$$

**Figure 9:**
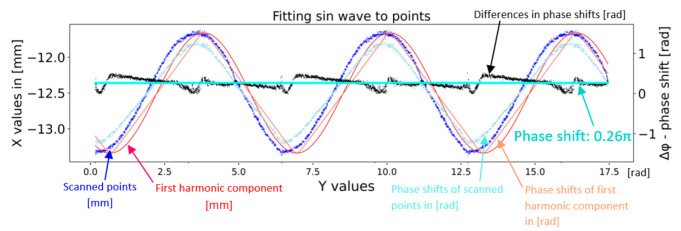
Fitting the computed first harmonic frequency and compensation sinusoidal wave.

Although the Fourier transform is a universal and reliable method, the output from this method does not generate sufficiently accurate values. For this reason, the first harmonic component is defined according to the angular velocity sensing parameter given in Table 2. The justification for the use of the Fourier transform is to obtain the phase shift of the first harmonic component. This is considered the first step in the calibration to remove the eccentricity that occurs in the data. The second step is the exact location of the simulated or calculated sinusoids to the actual recorded data. An example is shown in Figure 9. The scanned points are displayed in blue, and the first harmonic component is displayed in red, defined on the left vertical axis. The fitting of sinusoidal waves can be expressed as the average shift in the phase shifts of the individual points of the respective sinusoid (measured points and the first harmonic component). The phase shifts are calculated as the arc sine of each point. The offset of the measured points is shown in Figure 9 in light blue, and the displacement of the red sinusoid dots is shown by the orange dots. The difference between these shifts is shown by a black curve, which is averaged and shown in cyan color, i.e., by a phase shift of 0.26π of the first harmonic component of the Fourier transform from the real measured points.

**Figure 10:**
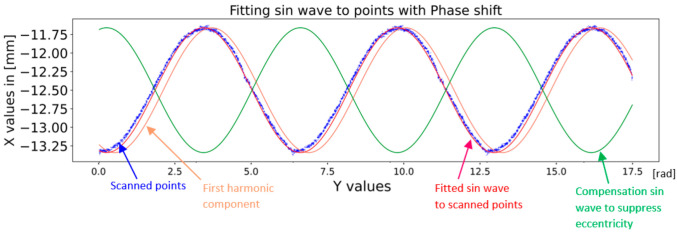
Fitted sine wave and compensation sine wave.

A summary of the results from previous calculations is shown in Figure 10. The original sinusoid of the first harmonic component of the Fourier transform is shown in orange. A fitted sine wave with a phase shift of 0.26π is shown in red. The eccentricity-suppressing compensation wave with a phase shift of π in radians is shown in green color in Figure 10.

**Figure 11:**
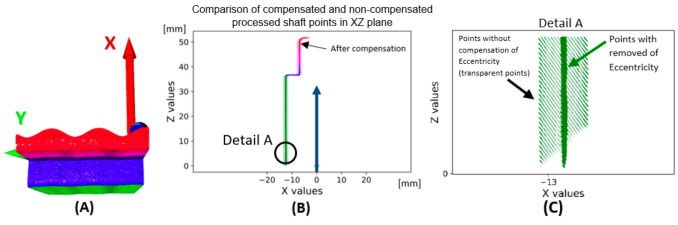
Data with suppressed eccentric: (**A**)—3D; (**B**)—projected point cloud to 2D, plane XZ; (**C**)—detail to points with removed first harmonic component as eccentricity (dark green color) and points with eccentricity (transparent green points).

**Figure 12:**
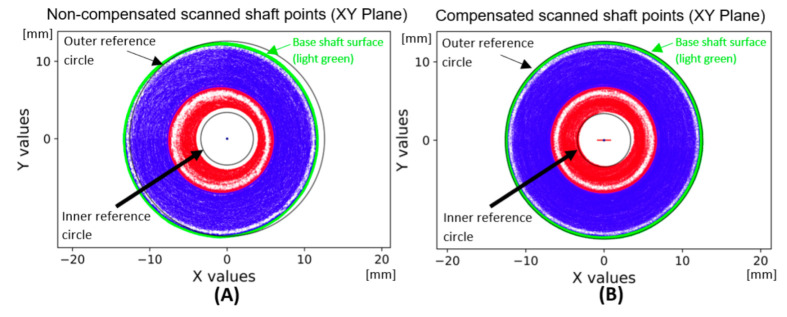
Comparison of rotated flatted surfaces scanned shaft to the real shape of shaft: (**A**) without compensation of eccentricity; (**B**) with compensation of eccentricity.

The application of the compensation wave to the data is shown in Figure 11. The base area of the shaft shown in green is cleaned of eccentricity. Subsequently, the eccentricity is manifested in the upper part of the shaft surface parallel to the plane XY. A comparison of uncompensated and compensated data is shown in Figure 12. The data ($D_{r}$) is transformed by the transformation matrix given in Equation (10), wherein the input data is in the form of a 3-dimensional matrix **K** defined in Equation (5) and a number *n* expressing the number of rotations recorded in radians. The value of $y_{max}$ is the maximum value occurring in the matrix **Y**. In the case of uncompensated data, the eccentricity is visible through the outer reference circle. In the compensated data, the unification of the shaft base surface with the outer reference circle is visible.

(10) $$D_{r}=\left[\begin{array}{c} X_{r}\\ Y_{r}\\ Z_{r} \end{array}\right]=\left[\begin{array}{ccc} \cos\left(n\pi \frac{Y}{y_{max}}\right) & -\sin\left(n\pi \frac{Y}{y_{max}}\right) & 0\\ \sin\left(n\pi \frac{Y}{y_{max}}\right) & \cos\left(n\pi \frac{Y}{y_{max}}\right) & 0\\ 0 & 0 & 1 \end{array}\right]*\left[\begin{array}{c} X\\ Y\\ Z \end{array}\right]$$

## 3. Results

**Figure 13:**
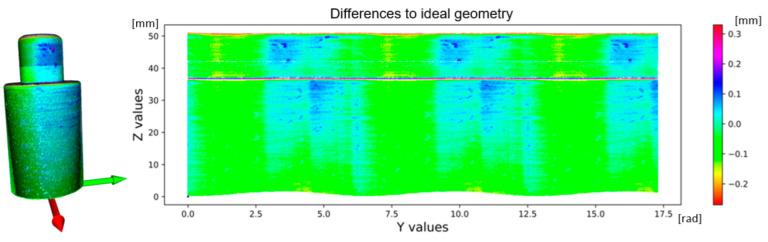
Illustration points to displacement from the nominal shape of the shaft. Left-side—3D visualization scanned shaft; right-side—flatten surfaces of scanned shaft.

**Figure 14:**
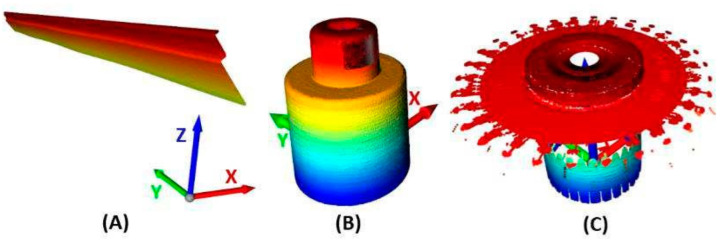
Illustration of application laser sensor in inspection gear wheel, where (**A**) is a raw point cloud of the scanned shaft, (**B**) is performed calibration for data of scanned shaft, and (**C**) is a processed scan of gear wheel according to calibration.

The primary goal of the process described in this article is to establish an automated calibration system focused on two primary tasks. The first is to convert points from the sensor coordinate system labeled S to the shaft coordinate system labeled C. The second is to record and remove the first harmonic component in the data, caused by the eccentricity between the shaft axis location and the rotary table axis. An eccentricity with an amplitude of 878.9 μm was recorded. The cleaned data are shown in Figure 13, where 90% of the data is in the range of ±100 μm. This image illustrates the second harmonic component corresponding to the cylindricality of a given part of the sensed shaft. Figure 14 shows a process based on shaft sensing and calibration automation. The correction parameters are generated and used in transforming the scanned gear point cloud to perform an inspection task of checking the correct geometric shape of the gear and finding any defects on the surface of these gear wheels.

### SW Parts of the Inspection System

**Figure 15:**
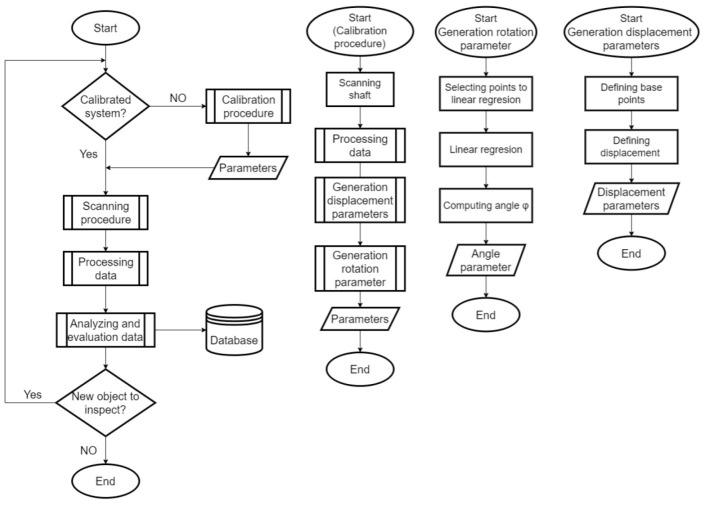
Algorithms of the inspection process.

The inspection system includes operations, as shown in Figure 15. There are functional parts that precede the experimental work, as has been mentioned. The SW solution consists of three basic blocks performing calibration functions, motion control, and sensor data processing. Calibration is the most comprehensive part of the software solution. For this reason, the improvement of calibration techniques and their implementation in the inspection represents a major contribution to the automation of the inspection.

## 4. Discussion

**Figure 16:**
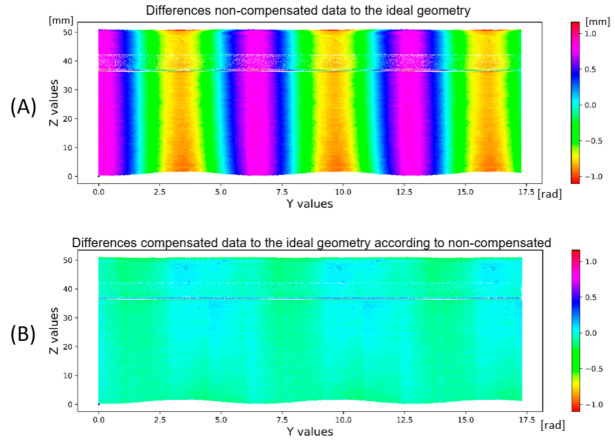
Impact of the first harmonic component in data: (**A**)—including the first harmonic component, and (**B**)—with the first harmonic component in data removed.

The design of this system is aimed at implementing new methods based on the development of sensory technology capable of creating 3D images of scanned objects and, based on their ability to design innovative solutions, shorten and minimize preparatory work and efforts to automate more sophisticated tasks in practice. For subsequent evaluation of the processed data of a specific inspection task or measurement analysis or finding defects, it is necessary to design new methods of deep learning due to the complexity of recorded data and variations of variables that reduce data quality. The result of the eccentricity removal in an automated manner is shown in Figure 16.

Another goal will be the design of automated positioning technology for 3D laser scanners and the possibility of unifying multiple images from different laser scanners to further streamline inspection procedures.

**Figure 17:**
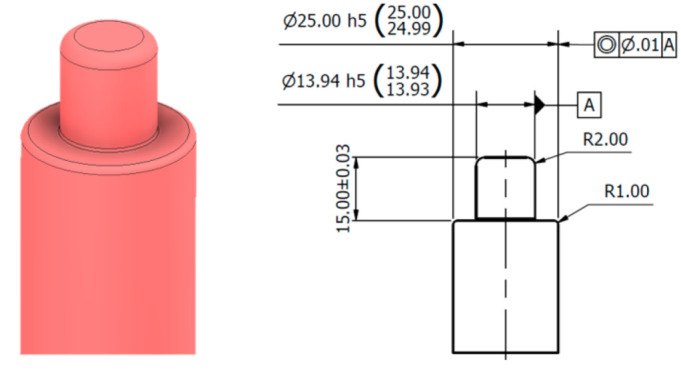
Design of calibration shaft according to measuring.

The actual state of work deals with the term of automated and non-automatic solutions due to complications in points defining the base shaft surface (Figure 17). Upon first view, it is a trivial issue, but as learned through solving many complex situations, it is very complicated to develop a resistant and full-cognitive solution. Suitable solutions should be in machine learning algorithms such as clustering methods, but it is necessary to explore this issue more deeply. The second limitation of this study is the suppression of the parallelism of the laser beam (line) with the shaft axis. It was suppressed by setting up the device before scanning and dealing with the fixation device, which will replace the adjustable arm.

## 5. Conclusions

This paper provides a brief description of the gear inspection system and a detailed description of the automated calibration system solution. The basic pillar of the system is using laser sensors and their ability to 3D scan controlled surfaces with resolution in units of micrometers, as shown in Table 1. The basic rationale is based on the diversity of shapes and dimensions of gears and their functional surfaces, where unique hardware settings are required to record functional surfaces or defined surfaces. Calibration is required for each change in the position of the laser sensor. This is due to the change in the mutual position of the S and C coordinate systems.

In the Introduction, the methods of solving sine wave fitting have been mentioned. This paper describes a sine fitting based on a method using a numpy library, which is a well-paralleled computing method suitable for multicore CPUs.

A rotating shaft made of additive technology was used for calibration, where the tolerance bands are in the tenths of a millimeter, as seen in Figure 13. Due to the scanning capabilities of the laser sensor used, it is more convenient to use a shaft made with high accuracy, as shown in Figure 17. It is due to the 2nd harmonic component in the data where repetitive patterns are visible in the visualization of differences between Figure 13 and Figure 16B.

## Figures and Tables

**Table 1:** Parameters of a laser sensor.

Laser Sensor	Parameter
Start of measuring range	70 mm
End of measuring range	120 mm
Resolution (*Z*-axis)	4 μm
Scanning points	640

**Table 2:** Scanning parameters.

Scanning Parameter	Value
Angular velocity [rad·s^−1^]	$\frac{1}{10}\pi$
Scanned frequency [Hz]	200
Number of profiles in one scan	11,000

## Data Availability

The data presented in this study are available upon request from the corresponding author.
